# Community social environments and cigarette smoking

**DOI:** 10.1016/j.ssmph.2022.101167

**Published:** 2022-07-16

**Authors:** Justin T. Denney, Gregory Sharp, Rachel Tolbert Kimbro

**Affiliations:** a Department of Sociology, Washington State University, USA; b Department of Sociology, Dartmouth College, USA; c Department of Sociology, Rice University, USA

**Keywords:** Cigarette smoking, Social cohesion, Neighborly exchange, Perceived danger, Activity space

## Abstract

Cigarette smoking remains a primary contributor to health disparities in the United States, and significant evidence suggests that smoking behavior is socially influenced. Though residential neighborhoods are important for health disparities, recent evidence suggests that people spend the majority of their waking time away from the residential neighborhood. We advance research on neighborhoods and smoking by using individual, neighborhood, and activity space data for adults in the Los Angeles Family and Neighborhood Survey (L.A.FANS). Moving beyond socioeconomic indicators of neighborhoods, we investigate the ways in which residential neighborhood social cohesion, neighborly exchange, and perceived danger impact smoking behavior after accounting for confounding factors in both the residential neighborhood and other activity spaces in which adults spend their days. We find that perceptions of danger in the residential neighborhood is robustly associated with the likelihood of smoking cigarettes. Further, measures of community social organization interact with perceived danger to influence smoking behavior. Adults with high levels of perceived danger are twice as likely to smoke if residing in communities with lower levels of social organization in the form of helpful, trusting, and supportive relationships. Understanding how the social organization of communities contributes to smoking disparities is important for curbing smoking's impact on population health.

## Highlights

• Adults with high perceived neighborhood danger are more likely current smokers.
• Adults in high danger low social cohesion neighborhoods twice as likely to smoke.
• Adults in high danger low neighborly exchange neighborhoods twice as likely to smoke.

## 1 Introduction

Cigarette smoking remains one of the most important causes of ill health and early mortality in the United States. Despite the fact that over the last three decades smoking rates declined precipitously, in part because of restrictions placed on where individuals could smoke, over 15% of the U.S. adult population continues to smoke cigarettes. Indeed, smoking related morbidity claims nearly 500,000 lives per year (Jamal et al., 2018).

Smoking is much more common among more socioeconomically disadvantaged populations (Pampel et al., 2010). To illustrate, over 60% of all smokers have a GED, high school, or less education and over a quarter are living below the poverty line (Jamal et al., 2018). Differences persist by racial and ethnic identity as well. Roughly the same proportion of Blacks and Whites smoke (16%) while fewer Asians and Hispanics (roughly 10%) and more Native Americans (32%) do so (Jamal et al., 2018).

Neighborhood environments have been shown to contribute to poor health in several adult health studies, including smoking (Kawachi & Berkman, 2003; Kravitz-Wirtz, 2016). Karasek et al. (2012) find that neighborhood social norms shape smoking behavior. And social cues and smoking-friendly environments are much more common in more disadvantaged neighborhoods (Jahnel et al., 2018). Indeed, the tobacco industry has long targeted advertising to lower income populations (Brandt, 2007) and this appears to have negatively impacted disadvantaged places for decades. Beyond socioeconomic considerations, scholars have begun to establish relationships between cigarette smoking and the ways in which communities are organized by other social characteristics. For example, research has reported lower likelihoods of smoking among residents in more socially cohesive neighborhoods characterized by high levels of trust, helpfulness, and connectedness among fellow neighbors (Echeverría et al., 2008; Holmes & Marcelli, 2014).

Similar to other health outcomes, however, the substance use and neighborhood effects literature often reveals inconsistent findings and modest results, particularly when stratified by individual sociodemographic characteristics (Karriker-Jaffe, 2011; Kravitz-Wirtz, 2016). Scholars have pointed to the lack of attention paid to temporal and spatial dynamics of neighborhoods as key contributors to these mixed findings (Inagami et al., 2007; Jones & Pebley, 2014; Kimbro et al., 2017; Sharp et al., 2015; Sharp & Kimbro, 2021). An overarching takeaway from existing activity space work is that contextual effects on health will vary across space and time; thus, residential neighborhood effects may be confounded by daily exposures to non-residential characteristics, and not considering non-residential exposures may, in turn, overestimate the influence of residential neighborhood effects on health (Chaix et al., 2017; Vallée et al., 2015). Given that recent evidence finds that people tend to spend the majority of their waking time away from the residential neighborhood (Browning et al., 2021; Wang et al., 2018), accounting for activity space exposures may clarify associations between residential neighborhood conditions and smoking behavior.

In this article, we employ novel longitudinal data from the Los Angeles Family and Neighborhood Survey (L.A.FANS) to contribute innovative insights to how and why neighborhoods matter for cigarette smoking. To our knowledge, there are no studies that construct exposure-weighted measures of context to systematically assess the relative impacts of place on adults’ smoking reports. We also update the extant literature by investigating the ways in which neighborhood social organization (i.e., social cohesion, neighborly exchange) may buffer the negative effects of community stressors, such as perceived lack of safety or fear, to reduce the risk of smoking cigarettes.

## 2 Neighborhood social environments and cigarette smoking

Earlier research on neighborhoods and cigarette smoking consistently links living in more socioeconomically deprived areas with increased risk of smoking (Chuang et al., 2005; Diez Roux et al., 2003). More recent work finds similar relationships between neighborhood SES and smoking, further elaborating on the relationship. For example, Kravitz-Wirtz (2016) finds that neighborhood poverty matters for smoking initiation, but only for White residents and only once the duration of poverty exposure is considered. In other words, prolonged poverty is associated with smoking initiation for some adults. In a recent study based in Seattle, WA and focused on adults age 30 to 39, Cambron et al. (2019) similarly show an elevated cigarette smoking risk for adults in higher poverty neighborhoods.

### 2.1 Moving beyond neighborhood SES

More research is needed to understand the linkages between neighborhood social environments and cigarette smoking. Neighborhood and community life has the potential to organize the ways in which individuals think about and behave toward health. Community social organization can take many forms and be measured in several different ways. As examples, social cohesion, or the general feeling of closeness to others in one's neighborhood; neighborly exchange, indicated by how often residential neighbors do favors or give advice to each other; and perceptions of neighborhood danger and safety are indicators of how and to what extent an individual trusts those around them and feels comfortable and safe in their surroundings (Bjornstrom, 2011; Bjornstrom & Kuhl, 2014; Carpiano, 2008).

There are independent and potentially competing ways in which community social organization might impact smoking. On the one hand, the stress paradigm (Pearlin, 1989) suggests that disadvantage, both at the individual and community level, impacts the capacity to cope. This may give rise to some unhealthy behaviors, such as cigarette smoking, as a means to deal with stressful circumstances (Pampel et al., 2010). Though stress mechanisms are well established in terms of socioeconomic conditions, stressors may also be influenced by how well connected individuals are with the neighborhoods they are embedded in, how safe they feel in those neighborhoods, and how closely linked residents are in terms of social status in relation to others in the community (Stockdale et al., 2007).

On the other hand, smoking is a social activity and one that is more prevalent and supported in more pro smoking social networks (Smith & Christakis, 2008). In a recent study, Blok et al. (2017) report that the smoking behavior in an individual's family and friend social network is a strong predictor of whether a smoker quits smoking and whether a smoker relapses after cessation. Places, including residential neighborhoods, where individuals feel connected and safe, may encourage prevalent and visible behaviors, such as cigarette smoking.

Several recent studies bear this out. Carpiano's (2008) important work on female primary caregivers finds that increased access to community information that might ease stressors related to employment and child care is directly linked with a lesser probability of smoking. The work of Reitzel et al. (2013) demonstrate that social cohesion, indicated by self-reported trust and connectedness between neighbors, facilitated smoking cessation in communities with strong interpersonal connections between residents. Other studies have found that higher neighborhood social cohesion associates with more successful cessation attempts, less smoking relapse, and an overall lower likelihood of being a current smoker (Fleischer et al., 2015; Holmes & Marcelli, 2014; Patterson et al., 2004).

It is also likely that neighborhood social organization dynamics may interact with social stressors to shape smoking behavior. Existing research has demonstrated how neighborhood social support mechanisms (e.g., social cohesion, neighborly exchange) may buffer the deleterious impacts of stressful circumstances, including heightened perceptions of crime and danger, as well as other disadvantaged conditions on smoking and various health outcomes. For example, there is evidence that residing in neighborhoods with greater levels of social cohesion mitigates the negative effects of personal and neighborhood stressful conditions on mental health (Choi & Matz-Costa, 2018; Dawson et al., 2019; Kingsbury et al., 2020). Research also finds higher neighborhood satisfaction to be protective of the unhealthy effects of high perceptions of crime on blood pressure (Coulon et al., 2016). Another recent study shows that neighborhood social cohesion buffers the cumulative detrimental impacts of exposure to discrimination on reduced telomere length—a physiological measure of wear and tear on the body—over time (Hailu et al., 2022). Thus, if perceived danger coincides with living in neighborhoods with high levels of social cohesion or neighborly exchange, having strong community engagement and connectedness could be stress-relieving and dampen the likelihood of smoking. In turn, residents of neighborhoods characterized by low levels of social cohesion or neighborliness who also feel unsafe in their neighborhoods may be more apt to smoke cigarettes as a coping mechanism (Pearlin, 1989).

Individuals may also feel either connected to, or an outsider in, their community through their racial and ethnic identity. Pickett and Wilkinson (2008) review a series of studies for group density impacts on well-being. Co-ethnic density refers to the concentration of people of the same racial/ethnic group living in the same geographic area, such as the residential neighborhood. Some of the studies reviewed have linked better mental and physical well-being for individuals in communities with higher prevalence of their same racial/ethnic identity (Pickett & Wilkinson, 2008). Low co-ethnic density may lead to decreased attachment to community, possibly associating with neighborly distrust and community disorganization and dissatisfaction (Dekker, 2012; Sharp, 2019). Similar to other kinds of neighborhood disadvantage, low co-ethnic density may operate through these mechanisms to increase poor health behaviors such as cigarette smoking. Or, motivated by the strong social networks perspective and smoking as noted above, high neighborhood co-ethnic density could lead to a shared sense of acceptance of smoking behavior among residents.

### 2.2 Incorporating both residential and non-residential places

Innovative recent studies examining the impact of place-level characteristics have moved beyond the characteristics of residential neighborhoods to examine the role of other activity spaces. This work has clarified links between place and health, showing that features of activity spaces, in combination with characteristics of residential neighborhoods, associate with health outcomes ranging from self-rated health to diabetes (Kimbro et al., 2017; Sharp et al., 2015; Sharp & Kimbro, 2021). A recent study of cigarette smoking by Shareck et al. (2016) shows that the likelihood to smoke is greater for young adults exposed to a higher density of residential and activity space tobacco retailers, compared to young adults who spend time in places with lower tobacco retailer presence. Importantly, there is also evidence to suggest that not accounting for the places in which people go outside the residential neighborhood may be a source of confounding that biases or misestimates residential effects on health (Chaix et al., 2017). This research illustrates the importance of adjusting for non-residential characteristics and the amount of time spent in these and in residential environments when examining the impact of places on health outcomes and behaviors.

## 3 Objectives

In the current analysis, we aim to illuminate relationships between dimensions of community social organization, co-ethnic density, and cigarette smoking for Los Angeles adults while adjusting for important individual and community level covariates. Our first aim is to establish adjusted independent associations between our social organization measures, co-ethnic density, and smoking. Second, we examine if and to what extent neighborhood social cohesion and neighborly exchange interact with adults’ perceptions of danger in their neighborhood to impact smoking.

## 4 Data and methods

### 4.1 Data sources

This paper uses longitudinal data from the Los Angeles Family and Neighborhood Survey (L.A.FANS). L.A.FANS is a multistage, multilevel survey based on a stratified random sample of 65 census tracts in Los Angeles County and was conducted in two waves: Wave 1 in 2000–2002 and Wave 2 in 2006–2008. In Wave 1, L.A.FANS randomly sampled 65 census tracts in Los Angeles County stratified by poverty level: very poor (tracts in the 90th or above percentile); poor (tracts in the 60-89th percentiles); and nonpoor (tracts below the 60th percentile). In the final sampling stage, 50 households were randomly selected from a list of all households within sampled census blocks (households with children under 18 were oversampled) and in-person interviews were conducted with adults and children living in over 3000 households across the 65 sampled tracts (Peterson et al., 2004). In Wave 2, L.A.FANS tried to re-interview all respondents in the original sample, in addition to interviewing a sample of newcomers to each tract, but in-person interviews with health-related questions were only administered to those who remained in L.A. County (Peterson et al., 2011). Of the 1187 adult respondents interviewed in Wave 2, 34 respondents did not report an activity space location and 22 had missing data on any of the analysis variables, resulting in 1131 adult respondents. We structure the data longitudinally such that each observation represents one person-period, yielding a final analytic sample of 2262 person-periods.

L.A.FANS provides panel weights, which are a combination of the Wave 1 design weight and a Wave 2 attrition adjustment. These weights account for the oversampling of poor census tracts and households with children, and the attrition of eligible Wave 1 respondents due to non-response. The attrition weight is derived from the inverse of the predicted probability of non-response from logistic regression models. Panel weights are also designed to make the sample representative of the adult population of L.A. County at Wave 1 who reside in the county at Wave 2. In comparison to panel respondents, adults who left the panel tend to have less children, education, and income, and are less likely to be employed or a homeowner.

L.A.FANS also provides census tract identifiers based on respondents' place of residence and several of their regular activity locations. More specifically, L.A.FANS interviewers asked respondents to report the locations of their current workplace, grocery store, place of worship, and where they receive healthcare. Respondents could report up to three locations in Wave 1 and up to four in Wave 2. For each activity location, respondents provided either addresses or cross-streets, which were then geocoded by L.A.FANS staff (Peterson et al., 2011). Tract-level data on adult's racial/ethnic and socioeconomic environments are extracted from Census 2000 and the 2005–2009 American Community Survey and are appended to the Wave 1 and 2 respondent-level data, respectively. All census tracts have been normalized to 2000 boundaries.

### 4.2 Measures

The dependent variable, *smoking status*, is captured by three dichotomous indicators: 1) never smoked (reference), 2) former smoker, and 3) current smoker. More specifically, respondents are assigned to the current smoker category if they answered affirmatively to the survey question, “Do you smoke cigarettes?”, while those who answered “yes” to the question, “Did you ever smoke cigarettes?”, assigned to the former smoker category, with all other respondents assigned to the never smoked category.

Key to this analysis is adjudicating the effects of several measures representing dimensions of neighborhood social organization, including neighborhood *social cohesion* and *neighborly exchange*. Social cohesion captures the extent to which residents feel a general closeness within the neighborhood in terms of mutual trust and willingness to help each other (Bjornstrom & Kuhl, 2014; Carpiano, 2008). Specifically, five L.A.FANS questions tap whether respondents perceive their neighborhood as close-knit, trustworthy, helpful, amicable, and sharing common values. Responses are based on a Likert-type scale ranging from (1) “strongly agree*”* to (5) “strongly disagree” and items are reverse-coded so that higher values reflect stronger cohesion. Neighborly exchange is based on three questions tapping the frequency of contacts with neighbors that involve doing favors, giving advice to each other, and being vigilant of each other's property when left unattended. A fourth question asks about the number of neighbors the respondent talked with for at least 10 minutes (Sharp, 2018, 2019). For the first three questions, responses range from (1) “often” to (4) “never” (reverse-coded), while the values of the last question are (1) “none”, (2) “1 or 2”, (3) “3 to 5”, and (4) “6 or more.” Table 1 displays the particular survey items that make up neighborhood-level social cohesion and neighborly exchange.

**Table 1:** L.A.FANS questions that make up neighborhood social cohesion and neighborly exchange measures.

Measure and Survey Questions	Response Range
Social cohesion	(1) strongly agree — (5) strongly disagree
1. "This is a close-knit neighborhood."*	
2. "People in this neighborhood can be trusted."*	
3. "People in this neighborhood do not share the same values."	
4. "People around here are willing to help their neighbors."*	
5. "People in this neighborhood generally do not get along with each other."	

Neighborly exchange	
1. "About how often do you and people in your neighborhood do favors for each other? For example, watch each other's children, help with shopping, lend gardening or house tools."*	(1) often — (4) never


2. "When a neighbor is not home, how often do you and other neighbors watch over their property?"*	(1) often — (4) never

3. "How often do you and other people in the neighborhood ask each other advice about personal things such as child rearing or job openings?"*	(1) often — (4) never

4. "In the past 30 days, how many of your neighbors have you talked with for 10 minutes or more?"	(1) none, (2) 1 or 2, (3) 3–5, (4) 6 or more


Note: *Indicates reverse-coded. L.A.FANS, Los Angeles Family and Neighborhoods Survey.

These social organization measures are derived via an “ecometric” approach often employed when creating aggregates of survey responses about respondents’ neighborhood perceptions and behaviors (Raudenbush & Sampson, 1999; Sampson et al., 1997). Specifically, we execute three-level item response models—items nested within individuals nested within tracts—and use the resulting empirical Bayes adjusted intercept (EB residuals) as the neighborhood social cohesion and neighborly exchange scores (e.g. Browning & Cagney, 2003; Carpiano, 2008). Take neighborhood social cohesion as an example. At level 1 (within-individual variation), the five items comprising the social cohesion scale are modeled as follows:

$$Y_{ijk}=\pi_{jk}+\sum_{p=1}^{5}\alpha_{p}D_{pijk}+e_{ijk}$$

where *Y*_*ijk*_ is the response to item *i* of person *j* in neighborhood *k*, *π*_*jk*_ is the intercept and can be interpreted as the respondent's latent social cohesion score, α_*p*_ refers to the item “difficulty,” *D*_*pijk*_ is a dummy variable coded as 1 if response *i* is to item *p* in the 5-item social cohesion scale and 0 otherwise, and *e*_*ijk*_ is the error component and is assumed to be independent and follow a normal distribution.

The level-2 equation (between-individual variation), which models respondents’ latent perceptions of social cohesion adjusted for a host of individual-level characteristics, can be written as follows:

$$\pi_{jk}=\beta_{0k}\sum_{q=1}^{13}\beta_{q}X_{qjk}+r_{jk},where\;r_{jk}∼N\left(0,\sigma^{2}\right)$$

where *ß*_0*k*_ is the intercept and is the ‘true’ score on social cohesion for neighborhood *k*; *X*_*qjk*_ represents a value for the respondent-level predictor *q* for individual *j* in neighborhood *k*, and *ß*_*q*_ is the effect (slope) of each *q* on individual *j*'s expected score, and *r*_*jk*_ is an independent, normally distributed error term with variance $\sigma^{2}$. This model adjusts for several individual-level covariates that may bias responses to the social cohesion items (see Carpiano, 2008), including age (years), gender (1 = female), race/ethnicity (Latino, non-Latino Black, non-Latino Asian/Other, non-Latino White), nativity (1 = foreign-born), marital status (1 = married), employment status (1 = employed), education (years), family income (logged), presence of children (1 = yes), length of neighborhood residence (years), perceived size of the neighborhood, which ranges from (1) “the block or street where you live” to (4) “an area larger than a 15-minute walk from your house.”

At level three (inter-neighborhood variation), the adjusted neighborhood intercepts are modeled as follows:

$$\beta_{0k}=\gamma_{000}+\mu_{00k},where\;\mu_{00k}∼N\left(0,\tau_{\beta }\right)$$

where $\gamma_{000}$ is the grand mean level of social cohesion, while *μ*_00*k*_ is a level-3 random effect and is assumed to be normally distributed with variance $\tau_{\beta }$. This random effect represents the deviation of each neighborhood's mean score from the overall social cohesion grand mean level. The subsequent standardized EB residuals are used as the final neighborhood social cohesion score, and account for differences in the reliability with which the neighborhood intercepts, $\beta_{0k}$, are estimated (Raudenbush & Bryk, 2002).

There are two neighborhood-level structural measures: *co-ethnic density* and *socioeconomic disadvantage*. Living in neighborhoods with other residents of similar ethnic backgrounds has been shown to be associated with better health, presumably via the dissemination of healthy norms, behaviors, and information (see Bécares et al., 2012). Co-ethnic density is the percentage of the tract population that is the same race/ethnicity as the respondent based on five groups: Latino, non-Latino White, non-Latino Black, non-Latino Asian, and non-Latino Other. Socioeconomic disadvantage is a commonly used measure of neighborhood SES (e.g. Xue et al., 2007) comprised of five variables (all percentages): individuals living below the poverty line, those in the labor force unemployed, households on public assistance, female-headed households with children, and adults 25 and older without a high school diploma.

Using L.A.FANS respondent data and following previous research (Kimbro et al., 2017), we use the amount of time spent in residential neighborhood and activity space locations to estimate exposure weights specific to each respondent. Though L.A.FANS does not provide the precise amount of time respondents engage in each activity, we can use survey responses to estimate the weekly average amount of time spent grocery shopping, attending religious services, and obtaining healthcare. With respect to workplaces, respondents reported up to three jobs, and provided the average number of hours worked per week for each job. For healthcare-related activities, respondents were also asked to recall how many times in the previous year they saw a doctor for an illness or injury, and how many times they saw a doctor for a physical examination or check-up. Each visit is assumed to last 2 hours; for example, if a respondent reported four healthcare visits in a month, they are allocated 2 hours per week to that activity tract. Similarly, respondents were asked to report the number of times they attended religious services in the past year. Respondents could report their attendance in per week, per month, or per year, and we converted per month and per year responses to the number of services attended per week. Again, each service attended is assigned 2 hours and then aggregated to weekly hours for each respondent. L.A.FANS did not ask respondents the number of times they went to grocery stores, so we assigned 2 hours per week for each store location provided, assuming that households average two 1-hour shopping trips each week (Institute, 2018).

To account for potentially long daily commute times by L.A. County workers, we excluded this time from our exposure weights. To estimate the number of hours spent commuting to work during a given week, we use the Census/ACS to retrieve average commute times (in minutes) to work by census tract and mode of transportation (drive, carpool, bus). This information is then linked to each L.A.FANS respondent based on their reported mode of transportation and home census tract. Commute time is then doubled and multiplied by five to reflect daily roundtrips and a five-day work week; thus, an average work commute of 1 hour is assigned 2 hours per day and 10 hours per week.

To arrive at the average hours per week spent in the respondents' residential neighborhoods, the total amount of time spent in their activity spaces and commuting to work is subtracted from the total hours per week (168). Final exposure weights for each residential and activity space neighborhood are computed by dividing the hours spent in each context by 168 hours. Respondents typically spend 78 percent of the time in the residential neighborhood in a given week, 20 percent in their activity space locations, and two percent commuting to and from work. By only accounting for workplace transportation, we acknowledge that we are likely underestimating adults’ total transportation time. Here, we assume that changes to exposure weights and subsequent findings would be negligible and that our substantive conclusions would hold. Note that sleep time is included in the denominator because existing research indicates that residents of disadvantaged neighborhoods have worse sleep (quality and quantity) than residents of more advantaged neighborhoods (Fuller-Rowell et al., 2016). Results were substantively similar with and without sleep time in the exposure weights calculation.

These exposure weights are then used to create residential and activity space exposure-weighted measures. For each respondent, we apply their exposure weight to their home and activity space measures to arrive at new weighted scores (e.g., residential social cohesion exposure). Consistent with prior research (Inagami et al., 2007; Kimbro et al., 2017; Sharp & Kimbro, 2021), each activity space exposure measure is a weighted average across all activity space contexts, reflecting individuals' overall activity space exposures rather than separate activity-specific measures. We take this approach because including separate activity space measures in our models induces a nontrivial amount of multicollinearity due to high correlations between the unique activity locations. By contrast, we find that correlations between individuals' residential exposure measures and their overall activity space exposure measures are reasonable. It should be noted that variables derived from census data (co-ethnic density, socioeconomic disadvantage) have both residential and activity space exposure measures, while neighborhood measures based on L.A.FANS data (social cohesion, neighborly exchange) only include residential neighborhood exposure versions. This is because these measures are based on L.A.FANS survey questions pertaining to the respondent's current neighborhood of residence, and there were insufficient sample sizes across activity space neighborhoods to create activity space social cohesion and neighborly exchange measures.

Following prior work on social organization in Los Angeles (see Sharp, 2018), individual *perceived danger* is a 4-item question tapping how dangerous respondents feel it is to walk around their neighborhood at night, including (1) “completely safe,” (2) “somewhat safe,” (3) “somewhat dangerous,” and (4) “completely dangerous.” Respondents who feel their neighborhoods are “somewhat” or “completely” dangerous are coded 1 and 0 otherwise.

We also include several individual-level control variables. Demographic characteristics are *race/ethnicity* (Latino, non-Latino Black, non-Latino Asian/other, and non-Latino White), *nativity* (1 = foreign born), *gender* (1 = female), *age* (years), *married* (1 = yes), and *presence of children* (1 = yes). Socioeconomic characteristics are *family income*, which is the sum of household earned and transfer income adjusted to 2007 dollars and transformed via the inverse hyperbolic sine (IHS) function, *education* (years of schooling), *employment status* (1 = employed), and *health insurance status* (1 = uninsured). Other covariates include *length of neighborhood residence* (years in the current census tract), and a binary indicator for *survey wave*. Table 2 presents weighted descriptive statistics of all variables used in the analysis.

**Table 2:** Weighted descriptive statistics for analysis variables, L.A.FANS Waves 1 and 2 (N = 2262).

	Mean/%	SD
Smoking status		
Never smoker	69.46	
Former smoker	16.41	
Current smoker	14.12	

Community social organizational characteristics		
Neighborhood social cohesion	0.27	1.03
Neighborhood neighborly exchange	0.17	1.18
Individual perceptions of danger (1 = yes)	21.68	
Neighborhood social structural characteristics		
Residential co-ethnic density	33.14	20.96
Residential socioeconomic disadvantage	0.02	0.77
Activity space co-ethnic density	5.54	6.91
Activity space socioeconomic disadvantage	−0.02	0.16

Individual-level covariates		
Age	45.00	15.78
Female	48.87	
Race/ethnicity		
White	38.14	
Black	7.73	
Latino	39.00	
Asian/Other	15.14	
Foreign born	46.76	
Married	52.35	
Presence of children	46.00	
Family income (thousands)	62.13	71.85
Education (years)	13.68	4.14
Employed	68.48	
Uninsured	23.17	
Length of neighborhood residence (years)	10.21	10.61

*Note:* L.A.FANS, Los Angeles Family and Neighborhood Survey; SD, standard deviation.

### 4.3 Analytic strategy

Recall that by creating exposure-weighted residential and activity space measures corresponding to the adult respondent (individual level), we structure our data longitudinally with time (survey wave) nested within individuals. As such, we estimate a series of multilevel multinomial logistic models predicting former smoker and current smoker versus the reference category never smoker. We execute four models: Model 1 includes neighborhood social cohesion and neighborly exchange measures. Model 2 adjusts for Model 1 variables and residential and activity space social-structural measures. Model 3 adjusts for Model 2 variables and includes individual perceptions of danger, and the full model (Model 4) adds the individual-level controls. We account for the correlated error structure in our data and report robust standard errors clustered at the individual level. For interpretation purposes, we convert logistic regression coefficients to average marginal effects (AMEs) and report 95% confidence intervals (CIs).

In addition, we investigate whether associations between neighborhood social cohesion, neighborly exchange, and smoking are shaped by respondent perceptions of danger in the neighborhood. To do so, we interact perceptions of neighborhood danger with neighborhood social cohesion and neighborly exchange, respectively. We enter these interaction terms in two separate, fully adjusted multinomial logistic models. Rather than rely on p-values associated with the interaction terms—a problematic approach to assessing statistical significance of interaction effects in nonlinear models (see Ai & Norton, 2003; Mize, 2019)—we illustrate the effects by plotting predicted probabilities of current smoker status with their respective 95% confidence intervals (see Mize, 2019). All analyses are executed using Stata 16 (StataCorp, 2019) and use L.A.FANS panel weights.

## 5 Results

Table 3 presents average marginal effects (AME) from multinomial regressions predicting the probability of being a current smoker compared with being a never smoker. Here, our focus is on current smoker status rather than former smoker status, and thus we do not present results predicting former smoker vs. never smoker (available upon request). The AMEs can be interpreted as the average change in the probability (expressed as percentage points) of being a current smoker, with corresponding changes in the independent predictors. For example, in Model 1, a one-standard-deviation increase in neighborhood social cohesion is significantly associated with a decrease (of 2.3 points) in the probability of being a current smoker (AME = −0.023, p ≤ 0.05), controlling for neighborly exchange. The social structural variables are added in Model 2 and explain the association between neighborhood social cohesion and current smoker status in Model 1. Similar to other research, Model 2 shows that higher levels of socioeconomic disadvantage in the residential neighborhood associate with a higher probability of being a current smoker (AME = 0.078, p ≤ 0.001). The associations between residential neighborhood and activity space co-ethnic density and smoking do not reach statistical significance in any model specifications.

**Table 3:** Estimates from multilevel multinomial logistic models predicting current smoker status (compared with never smoked), L.A.FANS Waves 1 and 2 (N = 2262).

	Model 1			Model 2			Model 3			Model 4		
	AME		95% CI	AME		95% CI	AME		95% CI	AME		95% CI
Community social organizational characteristics												
Neighborhood social cohesion	−.023	*	(-.044, −.001)	.008		(-.016, .033)	.011		(-.013, .036)	.010		(-.015, .034)
Neighborhood neighborly exchange	.000		(-.025, .025)	−.005		(-.030, .020)	−.006		(-.031, .019)	−.004		(-.031, .022)
Individual perceptions of danger (1 = yes)							.076	*	(.011, .141)	.067	*	(.011, .123)

Neighborhood social structural characteristics												
Residential co-ethnic density				−.004		(-.014, .007)	−.003		(-.013, .008)	−.001		(-.013, .011)
Residential socioeconomic disadvantage				.078	***	(.047, .109)	.063	***	(.032, .095)	.059	***	(.023, .095)
Activity space co-ethnic density				−.004		(-.036, .028)	−.004		(-.036, .028)	−.029		(-.064, .006)
Activity space socioeconomic disadvantage				−.030		(-.156, .096)	−.035		(-.161, .091)	−.031		(-.147, .085)
Individual controls included	No			No			No			Yes		

*Note:* L.A.FANS, Los Angeles Family and Neighborhood Survey; AME, average marginal effect; CI, confidence interval. Individual controls include age, gender, race/ethnicity, nativity, marital status, presence of children, family income, education, employment status, insurance status, and length of neighborhood residence. All models include survey wave. All contextual measures are exposure-weighted.

*p < 0.05; **p < 0.01; ***p < 0.001.

Model 3 of Table 3 shows that respondents who feel their neighborhoods are dangerous hold a higher probability of being a current smoker (AME = 0.076, p ≤ 0.001) than those who feel their neighborhoods are safe, adjusting for neighborhood social cohesion, neighborly exchange, and the social structure characteristics of adults’ neighborhoods and activity spaces. Finally, Model 4 adjusts for the full range of individual-level control measures. In this model, we see that the AMEs for perceptions of danger and residential neighborhood disadvantage are essentially unchanged and remain statistically significant. That is, our individual-level controls do little to impact the associations between perceived danger, neighborhood disadvantage, and smoking.

**Fig. 1:**
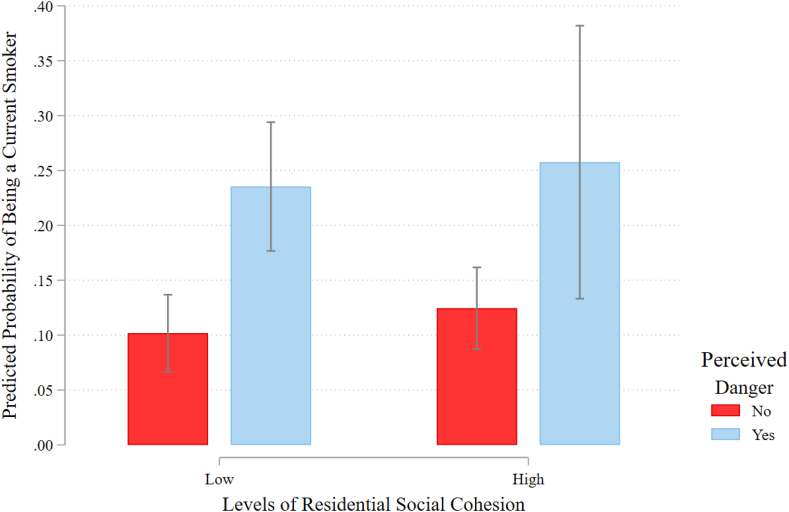
Predicted probabilities with 95% confidence intervals of being a current smoker by residential social cohesion and perceived danger.

**Fig. 2:**
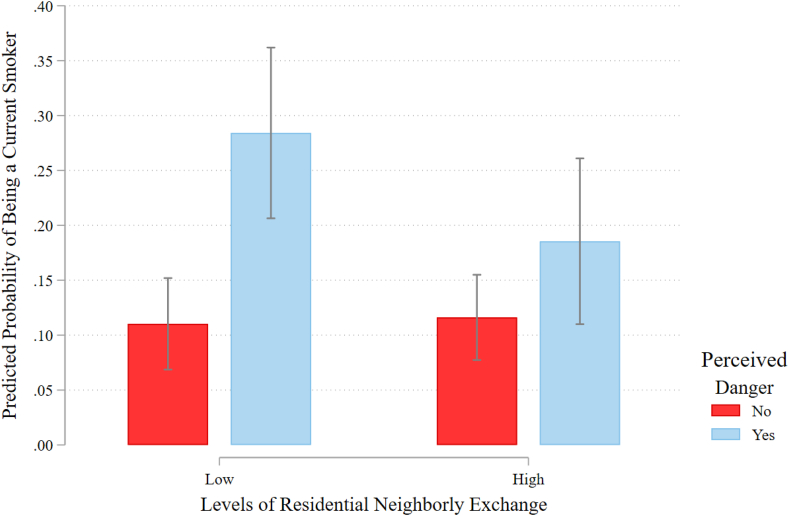
Predicted probabilities with 95% confidence intervals of being a current smoker by residential neighborly exchange and perceived danger.

To address our second objective, we interact our neighborhood social organization measures with individual perceptions of danger and present predicted probabilities with 95% confidence intervals in Fig. 1 and Fig. 2 (Mize, 2019). High and low levels of residential social cohesion and neighborly exchange represent one standard deviation above and below the mean, respectively. Beginning with Fig. 1, we see that among residents of neighborhoods with low levels of social cohesion, the predicted probability of being a current smoker is more than twice as high for individuals who feel their neighborhood is dangerous than their counterparts who feel their neighborhood is safe (predicted probabilities of 0.24 vs. 0.10, p ≤ 0.05). Adults who are fearful of their neighborhood surroundings are more likely to smoke than those who feel safe, even if their neighborhoods are highly cohesive, but this difference in predicted probabilities does not reach statistical significance.

Fig. 2 shows a similar pattern for the interaction between residential neighborly exchange and perceptions of danger. For adults living in neighborhoods characterized by low levels of neighborly exchange, those who perceive their neighborhood as dangerous are almost three times as likely to smoke than those who feel their neighborhood is safe (predicted probabilities of 0.28 vs. 0.11, p ≤ 0.05). Residing in neighborhoods with high levels of residential neighborly exchange narrows this disparity substantially (0.19 vs. 0.12) and is no longer statistically significant.

## 6 Discussion

In the present study, we focus on associations between neighborhood social environments and cigarette smoking that move beyond neighborhood socioeconomic conditions. This work also contributes new insights into the ways in which features of neighborhood social organization (social cohesion, neighborly exchange) buffer or exacerbate stressful circumstances that impact smoking behavior. We find that living in neighborhoods with higher levels of social cohesion is related to a lower likelihood of being a current smoker in unadjusted models. But this association is explained by residential and activity space measures of socioeconomic disadvantage and co-ethnic density. We also find that individual perceptions of danger in one's community are robustly associated with higher likelihoods of currently smoking.

To our knowledge, this is the first study to examine how neighborhood social cohesion and neighborly exchange moderate the association between perceived danger and smoking. We find that when residential neighborhood social cohesion or neighborly exchange are low, individuals who feel their neighborhoods are dangerous are more than twice as likely to smoke than those who feel their neighborhoods are safe. But this smoking gap by perceptions of danger is narrowed to nonsignificance at high levels of neighborhood social cohesion and neighborly exchange. That is, our evidence suggests that community social organization in the form of helpful, trusting, and supportive relationships may buffer stress processes associated with perceived danger and, likely, smoking behavior. These results are in line with existing work highlighting the protective attributes of neighborhood social organization dynamics, such as strong social cohesion, for unhealthy behaviors (Carpiano, 2008; Echeverría et al., 2008; Stockdale et al., 2007; Villalonga-Olives et al., 2020). Our work is innovative in that it directly tests the theoretical mechanisms implied in the neighborhoods and smoking literature, particularly whether dimensions of neighborhood social organization buffer stressful circumstances that elevate the risk of cigarette smoking. Here, we have examined neighborhood social cohesion and reciprocated exchange between neighbors, but future research would profit from expanding to other measures of contextual social organization, including community-wide participation in routine organizations (Altschuler et al., 2004), as well as the presence of neighborhood institutions that may facilitate the dissemination of services and information on heathy behaviors (Small, 2006), such as stress-relieving activities and smoking cessation programs.

Recent studies have shown that including measures of place representing the multiple venues where people live, work, and play (i.e., activity spaces) affords more precise estimates of associations between neighborhood characteristics and health (Inagami et al., 2007; Sharp et al., 2015), including smoking (Shareck et al., 2016). Accordingly, we adjust our place-based measures for the amount of time adults spend in these contexts, as well as include activity space measures of social structural characteristics important for health behavior. In doing so, we avoid a common peril of many neighborhood effects studies; namely, relying only on the residential neighborhood as the only relevant context for health and ignoring daily exposures to non-residential spaces (Chaix et al., 2017; Kwan, 2012). This is not a trivial concern, given the potential implications for place-based interventions. Chaix et al. (2017), for instance, illustrate this “residential effect fallacy” by illustrating that residential service interventions could be overestimated by a factor of three from the bias induced by not considering individuals’ routine exposures to non-residential places.

Our study has several limitations. First, because our data are based on a sample of Los Angeles County, our results cannot be generalized to other urban or rural areas in the U.S. and beyond. In addition, L.A.FANS does not provide a complete list of respondents’ daily activity locations, which could underestimate our exposure weights for activity space measures and thus provide more weight to the residential context. Researchers should prioritize the collection of innovative, theoretically grounded measures, including GPS tracking and ecological momentary assessments (EMAs) (Browning et al., 2021; York Cornwell & Goldman, 2020), as well as spatially relevant indicators of community social organization that capture the nuanced ways adults perceive and interact with their neighbors and neighborhoods locally and beyond.

Another limitation is our use of census tracts as proxies for our residential and activity space neighborhoods. Indeed, census tracts vary in size and shape, as do the places where people live and spend their time within and across tract boundaries, which could lead to measurement error in our residential and activity space exposure measures. Future studies should adopt spatially explicit measures that conceptualize exposure areas as individually perceived neighborhoods (Vallée et al., 2015), residence-based or street-network buffers (Chaix et al., 2017; Perchoux et al., 2016), and ecological networks (Browning et al., 2017).

## 7 Conclusion

Any approach to further elaborate on the association between cigarette smoking and community or neighborhood social organization should consider the most appropriate geographic scale. Equally important, future research in this area should consider possible differences in the ways places influence health outcomes for different population groups. Programs and policies focusing on smoking prevention can and should focus more on place-level strategies. Community physical and social improvements serve to connect members in meaningful ways that can build social capital and trust and reduce fear and stress. These same features can help reduce cigarette smoking.

Despite the clear negative implications for short- and long-term health, smoking remains a detrimental health behavior for substantial portions of U.S. adults. As with most health disparities, smoking is more common among disadvantaged groups. Understanding how the social organization of communities contributes to smoking disparities will be important for curbing smoking's impact on population health disparities.

## CRediT author statement

Justin Denney: Conceptualization, Methodology, Writing- Original draft preparation, Writing- Review & Editing. Greg Sharp: Formal analysis, Software, Data Curation, Visualization, Writing- Original draft preparation, Writing- Review & Editing. Rachel Tolbert Kimbro: Writing- Original draft preparation, Writing- Review & Editing.

## Ethical statement

Hereby, I, Justin Denney, consciously assure that for the manuscript, Community Social Environments and Cigarette Smoking, the following is fulfilled:

1) This material is the authors' own original work, which has not been previously published elsewhere.
2) The paper is not currently being considered for publication elsewhere.
3) The paper reflects the authors' own research and analysis in a truthful and complete manner.
4) The paper properly credits the meaningful contributions of co-authors and co-researchers.
5) The results are appropriately placed in the context of prior and existing research.
6) All sources used are properly disclosed (correct citation). Literally copying of text must be indicated as such by using quotation marks and giving proper reference.
7) All authors have been personally and actively involved in substantial work leading to the paper, and will take public responsibility for its content.
